# A ferromagnetically coupled Fe_42_ cyanide-bridged nanocage

**DOI:** 10.1038/ncomms6955

**Published:** 2015-01-06

**Authors:** Soonchul Kang, Hui Zheng, Tao Liu, Kohei Hamachi, Shinji Kanegawa, Kunihisa Sugimoto, Yoshihito Shiota, Shinya Hayami, Masaki Mito, Tetsuya Nakamura, Motohiro Nakano, Michael L. Baker, Hiroyuki Nojiri, Kazunari Yoshizawa, Chunying Duan, Osamu Sato

**Affiliations:** 1 Institute for Materials Chemistry and Engineering, Kyushu University, 6-1 Kasuga-koen, Kasuga, Fukuoka 816-8580, Japan; 2 State Key Laboratory of Fine Chemicals, Dalian University of Technology, 2 Linggong Road, 116024 Dalian, China; 3 Japan Synchrotron Radiation Research Institute, 1-1-1, Kouto, Sayo-cho, Sayo-gun, Hyogo 679-5198, Japan; 4 Graduate School of Science and Technology, Kumamoto University, 2-39-1 Kurokami, Kumamoto 860-8555, Japan; 5 Faculty of Engineering, Kyushu Institute of Technology, Kitakyushu 804-8550, Japan; 6 Research Center for Structural Thermodynamics, Graduate School of Science, Osaka University, Toyonaka, Osaka 560-0043, Japan; 7 Institute for Materials Research, Tohoku University, Katahira 2-1-1, Sendai 980-8577, Japan

## Abstract

Self-assembly of artificial nanoscale units into superstructures is a prevalent topic in science. In biomimicry, scientists attempt to develop artificial self-assembled nanoarchitectures. However, despite extensive efforts, the preparation of nanoarchitectures with superior physical properties remains a challenge. For example, one of the major topics in the field of molecular magnetism is the development of high-spin (HS) molecules. Here, we report a cyanide-bridged magnetic nanocage composed of 18 HS iron(III) ions and 24 low-spin iron(II) ions. The magnetic iron(III) centres are ferromagnetically coupled, yielding the highest ground-state spin number (*S*=45) of any molecule reported to date.

## Abstract (web summary)

One area of interest in the field of molecular magnetism is the development of high-spin molecules. Here, the authors report a cyanide-bridged nanocage consisting of 18 high-spin iron(III) ions ferromagnetically coupled through 24 low-spin iron(II) ions, with a ground state spin of *S*=45.

## Graphical abstract

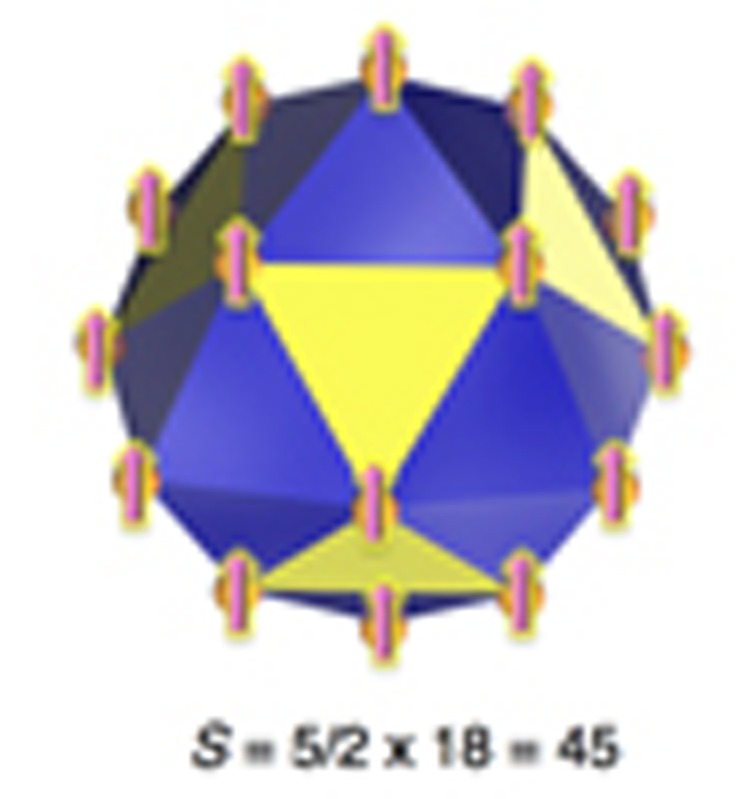

Self-assembled highly symmetric nanostructures are commonplaces in nature: for example, the icosahedra of virus capsids and the cuboctahedra of magnetite nanocrystals in magnetotactic bacteria. Following nature’s lead, attempts to prepare self-assembled discrete molecular architectures constructed through the coordination of metal ions and organic ligands such as metal–organic polyhedra have captivated many scientists’ attention^1^,^2^,^3^,^4^,^5^,^6^,^7^,^8^,^9^,^10^. The unique structural and electronic configurations, resulting from the combined features of both metal ions and organic molecules, can give way to diverse functional properties for many applications, such as molecular flasks^11^, catalysis^12^, ion channels^13^, sensors^14^, drug delivery systems^15^, nanomagnets^16^,^17^,^18^ including prototypes for quantum information processing^19^,^20^ and gas storage devices^21^,^22^,^23^,^24^.

One of the challenges in the field of molecular magnetism is to synthetically prepare new nanoarchitectures with high-ground state spin numbers. To date, numerous high-spin (HS) molecules have been reported^25^,^26^. Here, we report a giant-spin nanocage that contains 18 HS ferromagnetically coupled Fe^III^(*S*=5/2) ions resulting in a molecular ground state spin of *S*=45, the largest value known to date^27^. This nanoarchitecture is a mixed valent HS and low-spin (LS) cyano-bridged Fe^III-HS^_18_Fe^II-LS^_24_ compound, structured as a supramolecular cage with a nanometre-sized inner cavity space.

## Results

### Preparation of Fe_42_ cyanide-bridged nanocage

The strategy used to construct the magnetic nanocage is based on the preparation of metal–organic polyhedra^28^. We used metal–organic complexes as building blocks, which not only act as caps but also contain metal centres and cyano groups that enable the introduction of magnetic interactions. Namely, instead of organic tridentate pyridyl ligands, which can provide a large hollow polyhedral structure, we used monoanionic complex ligand {Fe(Tp)(CN)_3_}^−^ (Tp=hydrotris(pyrazolyl)borate) units^29^,^30^,^31^ as a trinucleating ligand of the metal ions for constructing magnetic nanocage. In addition, the choice of counter metal ions in these structures importantly enables the adjustment of their magnetic properties, facilitating the creation of HS ground states. We employed iron ions in consideration of the magnetic interaction and redox activity of the ferromagnetic metal–cyanide compound: Prussian blue Fe^III^_4_[Fe^II^(CN)_6_]_3_·*x*H_2_O (refs 16, 32). The reaction of Fe(CF_3_SO_3_)_2_, 1,3-di(4-pyridyl)propane (dpp), L-ascorbic acid and Li[Fe(Tp)(CN)_3_] in H_2_O led to the isolation of a new [Fe^III^_18_Fe^II^_24_] spin nanocage: [{Fe(Tp)(CN)_3_}_24_{Fe(H_2_O)_2_}_6_{Fe(dpp)(H_2_O)}_12_(CF_3_SO_3_)_6_]·18H_2_O (**1**·18H_2_O) as green cubic crystals (Supplementary Fig. 1), where **1** contains 42 iron ions, the largest number of metal centres in any cyano-bridged cluster reported to date^33^.

### Characterization of Fe_42_ cyanide-bridged nanocage

**Figure 1:**
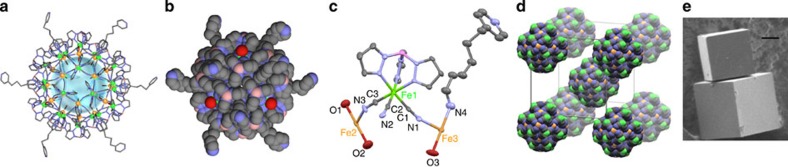
Crystal structure of 1·18H_2_O. Fe^II^ and Fe^III^ ions are shown as light green and orange balls, respectively. (**a**) A framework structure of a single [Fe_42_] nanocage: **1**·18H_2_O (14 Å diameter void, blue sphere). All the counter ions (CF_3_SO_3_^−^) and crystal solvent (H_2_O) are omitted for clarity. (**b**) As in **a**, but as a space-filling model; grey, blue, red, and pink spheres represent C, N, O, and B atoms, respectively. (**c**) The asymmetric unit of **1**·18H_2_O with thermal ellipsoids at 30% probability. Hydrogen atoms, counterions and solvent molecules have been omitted for clarity. (**d**) View of the crystal packing for cyano-bridged [Fe_42_] framework. (**e**) Scanning electron microscope image of crystals of compound **1**·18H_2_O, illustrating the cubic faces. A scale bar indicates 10 μm.

Crystallographic analysis of **1**·18H_2_O reveals that each octahedral {Fe(Tp)(CN)_3_} unit is connected to three Fe ions by three cyanide anions. The Fe ions are further ligated by water and dpp to give octahedral {Fe(NC)_4_(H_2_O)_2_} and {Fe(NC)_4_(dpp)(H_2_O)} coordination spheres, where the Fe centres are in a weakly distorted octahedral environment with axial water molecules (Fig. 1 and Supplementary Tables 1–3). Thus, 24 {Fe(Tp)(CN)_3_}, 12 {Fe(NC)_4_(dpp)(H_2_O)} and 6 {Fe(NC)_4_(H_2_O)_2_} building units are symmetrically disposed in an *O* space around a central point providing the cube cage, with a separation of 1.96 nm between the most distant Fe ions. Six severely disordered, charge-balancing trifluoromethanesulfonate anions are apparent outside the cationic [Fe_42_]^6+^ nanocages, keeping them well separated, and 18 solvent water molecules are located inside the cage.

The ^57^Fe Mössbauer spectrum measured for **1**·18H_2_O at 298 K can be deconvoluted into two doublets exhibiting quadrupole splitting (LS-Fe^II^:*δ* (isomer shift)=0.065 mm s^−1^, *Δ* (quadrupole splitting)=0.47 mm s^−1^; HS-Fe^III^: *δ*=0.43 mm s^−1^ and *Δ*=0.71 mm s^−1^) in a relative intensity ratio of Fe^II^/Fe^III^=0.55/0.45 Supplementary Fig. 2 and Supplementary Table 4). Charge considerations and Mössbauer spectroscopic measurements suggest that **1**·18H_2_O has 24 Fe^II-LS^ ions, to which the cyanide carbon atoms coordinate, and 18 Fe^III-HS^ ions at room temperature. Note that **1**·18H_2_O was prepared using {Fe^III^(Tp)(CN)_3_}^−^ as a starting material, where the cyanide carbon atoms are coordinated to Fe^III^ ions. Electron transfer from Fe^II^ ions to the {Fe^III^(Tp)(CN)_3_}^−^ units is, therefore, suggested to occur during the reaction, leading to an Fe^II-LS^–CN–Fe^III-HS^ linkage. Further, evidence of this linkage was obtained by synchrotron X-ray absorption spectroscopy (XAS) at the Fe L-edge. Comparison of XAS for **1**·18H_2_O with variants of the starting material compound, Li[Fe^III-LS^(Tp)(CN)_3_] and K_2_[Fe^II-LS^(Tp)(CN)_3_], infers that the valence composition of the 24 {Fe(Tp)(CN)_3_} is consistent with {Fe^II-LS^(Tp)(CN)_3_}^2−^ (See Supplementary Fig. 3, with further details of this measurement).

**Figure 2:**
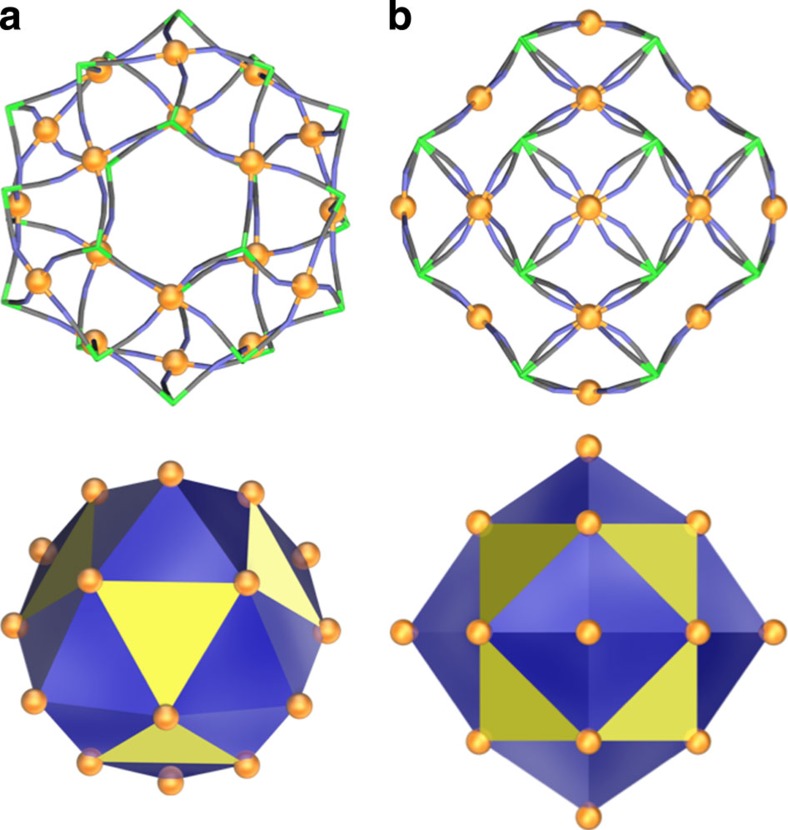
Crystal structure of Fe^III^ ions in compound 1·18H_2_O. Crystal structure looking down through a triangular window (**a**) and a square window (**b**). A skeleton structure with Fe^III^ atoms (orange balls) bridged by cyano groups of **1**·18H_2_O (above). Below each structure, Fe^III^ atoms have been extracted from the crystal structure. Note that the structure of **1**·18H_2_O is related to the stellated cuboctahedron structure. However, mathematically defined stellated cuboctahedron is stellated on every triangular face of the cuboctahedron (shown in yellow), whereas in **1**·18H_2_O only the square windows are stellated with 24 isosceles triangles (side lengths: 6.83, 6.83 and 7.85 Å).

The most remarkable structural feature of **1**·18H_2_O is that the 18 Fe^III-HS^ ions can be identified as the vertices of a highly symmetric entity shown in Fig. 2. First, 12 Fe^III-HS^ centres in {Fe(NC)_4_(dpp)(H_2_O)} units are defined as the vertices of cuboctahedron, all of whose sides have a length of 7.85 Å. Second, six square windows of the cuboctahedron are stellated with 24 isosceles triangles, each with side lengths of 6.83, 6.83 and 7.85 Å, with six vertices defined by Fe^III-HS^ ions from {Fe(NC)_4_(H_2_O)_2_} units^34^,^35^. The Fe^II^**–**CN**–**Fe^III^ linkage of **1**·18H_2_O classify it as a Prussian blue analogue^36^,^37^ from the standpoint of its electronic state. Prussian blue analogues exhibit in a molecule-based framework with cyanide-bridging akin to the three-dimensional Prussian blue compound Fe^III^_4_[Fe^II^(CN)_6_]_3_·*x*H_2_O. Prussian blue shows ferromagnetic behaviour with a Curie temperature *T*_c_ of 5.5±0.5 K (ref. 32). The observed ferromagnetism involves a long-range order of the Fe^III^ ions. While **1**·18H_2_O is much more complex than Fe^III^_4_[Fe^II^(CN)_6_]_3_·*x*H_2_O, the cyanide bridging units between the Fe^III^ ions are similar. The shortest Fe^III^**–**Fe^III^ distances for Prussian blue and **1**·18H_2_O are 7.2 Å and 6.8 Å through space and 10.2 Å and 9.9 Å along the Fe^III^**–**NC**–**Fe^II^**–**CN**–**Fe^III^ units, respectively. Accordingly, it is reasonable that the 18 Fe^III-HS^ (*S*=5/2) ions, at the vertices in **1**·18H_2_O, are coupled ferromagnetically.

### Magnetic properties of Fe_42_ cyanide-bridged nanocage

**Figure 3:**
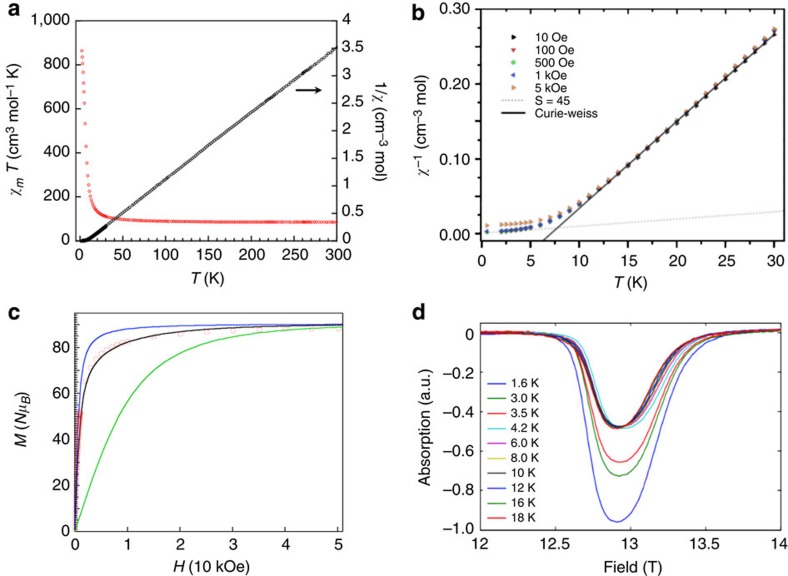
Magnetic characteristics for 1·18H_2_O. (**a**) Temperature dependence of *χ*_*m*_*T* and *χ*_*m*_^−1^ for **1**·18H_2_O (*H*_dc_=100 Oe from 2 to 30 K and *H*_dc_=10,000 Oe from 30 to 300 K). (**b**) *χ*^−1^ versus *T* for various applied fields. Calculations of susceptibility are represented below 5 K by a paramagnetic *S*=45 with a *g*=2, and the Curie–Weiss law above 10 K. (**c**) Magnetization versus external magnetic field curve for **1**·18H_2_O at 2 K. Red circles are experimental data. Blue line corresponds to simulation employing the Brillouin function for *S*=45 with *g*=2.0. The green line represents the 18 times value of the Brillouin function that corresponds to *S*=5/2 with *g*=2.0. The black line represents the simulation curve described in Methods. (**d**) The temperature-dependent EPR spectra of **1**·18H_2_O (360 GHz continuous wave EPR).

Figure 3a shows the magnetic properties of a polycrystalline sample of **1**·18H_2_O under a direct current field of 10 kOe from 300 to 30 K and 100 Oe from 30 to 2 K. It indicates the existence of predominantly ferromagnetic interactions and a resulting giant ground-state spin for **1**·18H_2_O. At 300 K, the *χ*_*m*_*T* product is 85.5 cm^3^ mol^−1^ K, and the data in the range 300**–**30 K can be fitted to the Curie**–**Weiss law, yielding *C*=83.2 cm^3^ mol^−1^ K and *θ*=+6.7 K. This *C* value is consistent with the expectations (78.8 cm^3^ mol^−1^ K with *g*=2.0) for 18 uncoupled Fe^III^ centres (*S*=5/2). On cooling, the *χ*_*m*_*T* value becomes slightly larger with temperature, abruptly increasing to 863 cm^3^ mol^−1^ K at 2 K (Supplementary Fig. 4). This magnetic behaviour and the positive Weiss constant suggest the existence of dominant ferromagnetic exchange interactions in **1**·18H_2_O. Figure 3b shows *χ*^−1^ versus the T-plot for various applied fields, which displays an inflection in *χ*^−1^ between 10 and 5 K as excited states depopulate, on further cooling below 5 K the slope starts to become linear again, suggesting that it is just the ground state that is mainly populated, following the dependence of a paramagnetic *S*=45, *g*=2.0, spin unit (dot line).

Moreover, the magnetization (*M*) at 2 K (Fig. 3c) rapidly increases at low fields, and then steadily increases with *H*>15 kOe to reach a near saturation value of 88.4 *μ*_*B*_ at 50 kOe, which is in good agreement with the expected value of 90 *μ*_*B*_ (with *g*=2.0) for a ground state of *S*_T_=90/2 (Supplementary Figs 5 and 6). This magnetization behaviour is significantly higher than the Brillouin curve corresponding to 18 non-interacting *S*_Fe_ spins (*S*=5/2, green line), fitting more closely the Brillouin curve for one *S*=45 centre (with *g*=2.0, blue line, see Methods). These data support the maximum possible spin state, *S*=45, which is the largest spin ground state number of any molecule ever prepared^27^.

To rule out the possibility of intermolecular interaction or a magnetic ordering between the molecule-based giant spins, electron paramagnetic resonance (EPR) spectra of **1**·18H_2_O have been examined. Figure 3d shows no evidence of intermolecular interaction is present within EPR on decrease of temperature to 1.6 K; the *g*=2.0 absorption increases in intensity with no significant variation of line width or shift in resonance field position to evidence intermolecular interactions. No evidence of anisotropy is observed in EPR measurements, it is reasoned that the geometry of the molecular structure of **1**·18H_2_O causes the cancelling of single ion anisotropic contributions. In addition, the temperature dependence of magnetization for **1**·18H_2_O under various applied magnetic fields does not show evidence of spontaneous magnetization down to 0.5 K (see Supplementary Fig. 7). These results indicate that the cyanide-bridged magnetic Fe_42_ nanocage exhibits the maximum spin ground state with isolated molecule *S*=45.

## Discussion

**Figure 4:**
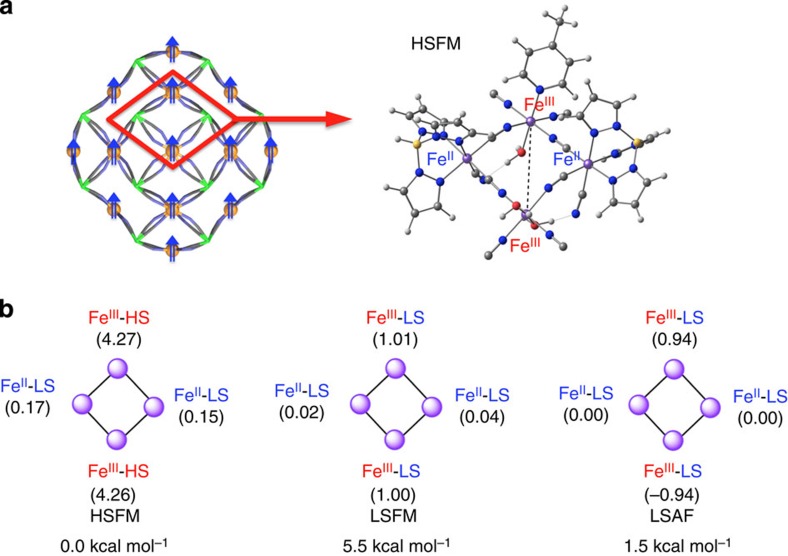
DFT calculation. (**a**) A skeleton structure of cyano-bridged [Fe_42_] framework. Fe^II^ and Fe^III^ ions are shown as light green and orange balls, respectively. Blue arrows indicate Fe^III^ sites that are ferromagnetically coupled. (**b**) Optimized geometry of the tetranuclear cyanide-bridged square complex in the HS ferromagnetic (HSFM) state, calculated spin densities, and relative energies of HSFM, LS ferromagnetic (LSFM) state and LS antiferromagnetic (LSAF) state at the B3LYP* level of theory. Units are in kcal mol^−1^. Computed energies of the tetranuclear cyanide-bridged square complex are 0.0 kcal mol^−1^ in the HSFM state (undecet state), 5.5 kcal mol^−1^ in the LSFM state (triplet state) and 1.5 kcal mol^−1^ in the LSAF state (open-shell singlet state). The HS antiferromagnetic (HSAF) state is not available as a low-lying open-shell singlet state. The distances between the diagonal Fe^III^ atoms are 6.817 Å in the HSFM state and 6.785 Å in the LSFM state.

DFT calculations were carried out to estimate changes in the electronic structures of **1**·18H_2_O. Calculations were simplified to a cyano-bridged molecular square^38^,^39^ formulated as [Fe^II^_2_**–**CN**–**Fe^III^_2_] with a Tp^−^ ligand on the Fe^II^ ion, a pyridine ligand, and H_2_O on the Fe^III^ ion (Fig. 4, and Supplementary Tables 5–7). Figure 4 shows HS ferromagnetic (HSFM) state was the ground state, and LS ferromagnetic (LSFM) and antiferromagnetic (LSAF) states were above 5.5 kcal mol^−1^ and 1.5 kcal mol^−1^, respectively. DFT calculations indicate the ferromagnetic character of a magnetic coupling between the two diagonal Fe^III-HS^ ions in the square framework. The calculated *J* value (35.5 cm^−1^ at the B3LYP* level) for the tetranuclear cyano-bridged square complex overestimates the magnitude of the exchange coupling parameters. The overestimation of coupling constants by a factor of 2–4 is not unusual in DFT calculations^40^,^41^. The calculation predicts the correct sign for *J* corresponding to the ferromagnetic nature of the ground state in the [Fe_42_] nanocage.

We have presented a Fe_42_ cyanide-bridged nanocage with a HS framework. Many metal–cyanide clusters have been synthesized since the discovery of Prussian blue. Our metal–cyanide polyhedron is the largest cyanide-bridged polynuclear cluster and exhibits a rare hollow structure. Among the various morphologies of nanoarchitectures, hollow spheres are of great interest because of their high surface to volume ratio and large pore volume, which could be exploited for promising applications in the controlled encapsulation and release of molecules.

In summary, we report a new high-nuclearity iron complex with a HS framework. In the Fe_42_ cyanide-bridged nanocage, magnetic metal centres are ferromagnetically coupled, yielding the highest ground state spin number (*S*=45) of any prepared molecule.

## Methods

### Synthesis of Fe_42_ cyanide-bridged nanocage

[{Fe(Tp)(CN)_3_}_24_{Fe(H_2_O)_2_}_6_{Fe(dpp)(H_2_O)}_12_(CF_3_SO_3_)_6_]·18H_2_O: **1**·18H_2_O.

A 1 ml aqueous solution of 17 μmol of Li[Fe(Tp)(CN)_3_] and 8.3 μmol of 1,3-di(4-pyridyl)propane was slowly layered over a 2 ml aqueous solution of 8.3 μmol of Fe(CF_3_SO_3_)_2_ and 1.7 μmol of L-ascorbic acid with H_2_O (1 ml) as a middle buffer layer under an aerobic condition. Crystallization required several weeks and gave crystals in 30% yield based on Li[Fe(Tp)(CN)_3_]. The crystals were washed with H_2_O and dried under reduced pressure for 12 h. As prepared compound, before vacuum drying, has approximately 50H_2_O molecules inside the cage (compound **1**·ca 50H_2_O), which become partially desolvated when exposed to air at room temperature. Therefore, the physical measurements performed on compound **1**·18H_2_O were prepared carefully to prevent desolvation. The reported structures have been characterized by the single-crystal X-ray crystallography (Supplementary Tables 1–3 and Supplementary Data 1 and 2).

### Elemental analysis

Elemental analysis for C_450_H_492_B_24_F_18_Fe_42_N_240_O_60_S_6_ (**1**·18H_2_O) is as follows: calculated (found) C, 40.45 (40.65); H, 3.71 (3.54) and N, 25.16 (25.30). Inductively coupled plasma atomic emission spectroscopy (ICP-AES) analysis was performed to measure the Fe content in the solid sample of **1**·18H_2_O. ICP-AES analysis shows that the Fe contents in **1**·18H_2_O is 16 wt%, which is in good agreement with the calculated value of 16.8 wt%. In addition, analysis of the sulfur content of **1**·18H_2_O with total sulfur analyser (TOX-100) reveals the sulfur content is 1.6 wt% (calcd. 1.4 wt%).

### X-ray structure determination

Single-crystal synchrotron radiation X-ray diffraction experiments were performed at 295 K for **1**·18H_2_O, and 100 K for **1**·ca 50H_2_O using a Rigaku Mercury2 CCD detector at BL02B1/SPring-8 (Hyogo, Japan). The wavelength of the incident X-ray was 0.6186 or 0.6202 Å. We used anomalous dispersion coefficients for structure refinement, *f*′ and *f*″, in dependence on X-ray energy calculated on the original *FPRIME* code of Cromer.

### SEM measurements

Scanning electron microscopy studies were performed with a Hitachi Ultra-high Resolution Scanning Electron Microscope SU8000.

### Physical measurements

Magnetic susceptibility measurements of samples were performed on a Quantum Design SQUID (MPMS XL-5 and MPMS XL-7) magnetometer. To prevent the loss of uncoordinated water molecules, the sample was introduced directly into the sample chamber at 100 K without purging, while flowing He gas. Keep the sample stay at 100 K for several minutes and then purge the chamber. At the end, vent the sample space with He gas and start the measurement. Data were corrected for the diamagnetic contribution calculated from Pascal constants. The Mössbauer spectra (isomer shift versus metallic iron at room temperature) were measured using a Wissel MVT-1000 Mössbauer spectrometer with a 57Co/Rh source in the transmission mode. All isomer shifts are given relative to α-Fe at room temperature. EPR measurements were performed with the Terahertz ESR Apparatus (TESRA-IMR) installed in the magnetism division of Institute of Materials Research (IMR), Tohoku University. A solenoid magnet is fed by a 90-kJ capacitor bank delivering a field pulse of 25 ms in width. Applied fields from 0 to 30 T were investigated for a range of EPR microwave frequencies from 90 to 405 GHz generated by backward-travelling wave oscillators. Measurements were performed in a He4 cryostat down to 1.6 K.

### Simulation of magnetization process

The magnetization curve was compared with the mean-field simulation assuming an HDVV (Heisenberg–Dirac–Van Vleck)-type spin Hamiltonian: *Ĥ*=−2*J*∑_(i,j)_*Ŝ*_*i*_·*Ŝ*_*j*_, where a uniform isotropic exchange parameter *J* makes all the pair of *S*=5/2 spins coupled. This Hamiltonian gives the energy eigenvalues *E*(*S*_total_)=−*J* {*S*_total_ (*S*_total_+1) −18*s*(*s*+1)} with the resultant spin of the molecule *S*_total_, and the magnetic susceptibility is easily calculated following the Van Vleck formula. The simulation curve with a ferromagnetic coupling *zJ*/*k*_B_=+0.5 K (*z*=17) and *g* factor of 2.0 agreed well with the experimental values.

### XAS measurements

X-ray absorption spectra were measured on the soft X-ray undulator beam-line BL25SU at Spring 8, Japan. Soft X-rays circularly polarized from a twin helical undulator were monochromated and focused onto a thin polycrystalline layer of **1**·18H_2_O. The measured sample was fixed with carbon tape to a sapphire sample holder. X-ray absorption spectra were measured by the total electron yield method in which the sample current is directly measured while scanning the photon energy. Measurements were performed at zero applied magnetic field and hence both positive and negative X-ray helices resulted in equivalent absorption spectra. The sample chamber for the soft XAS keeps a high vacuum of 10^−5^ Pa or the better. Measurements of **1**·18H_2_O were repeated incrementally over several days and were found not to exhibit changes in spectral line shape with respect to the time spent under vacuum. Care was taken during XAS measurements to control the effect of photoreduction. The incident beam intensity was incrementally reduced until consistent multiple measurements at the same sample spot were obtained after an intensity reduction to 7%. To ensure damage was not encountered in short time periods, rapid measurements over defined features of the L_3_ edge were measured repeatedly and ensured to be coincident.

### DFT calculation

Full details of computational method are given in the Supplementary Methods.

## Author contributions

T.L. and C.D. in Dalian and O.S. in Fukuoka conceived and supervised the project. S. Kang, K.H., S. Kanegawa and H.Z. planed, implemented materials synthesis and characterization and analysed the magnetic measurements data. K.S. finalized the synchrotron X-ray data. Y.S. and K.Y. contributed to the DFT calculation. S.H. performed the Mössbauer measurement. M.N. and M.M. contributed to analyses of the magnetic susceptibility. M.L.B. and H.N. were responsible for experiments performed in large magnetic fields and XAS measurements. T.N. contributed to the XAS experiments. S. Kang, Y.S., L.T. and O.S. wrote the manuscript. All authors discussed the results and commented on the manuscript. S. Kang, and H.Z. contributed to this work equally.

## Additional information

**How to cite this article**: Kang, S. *et al.* A ferromagnetically coupled Fe_42_ cyanide-bridged nanocage. *Nat. Commun.* 6:5955 doi: 10.1038/ncomms6955 (2015).

**Accession codes:** The X-ray crystallographic coordinates for structures reported in this Article have been deposited at the Cambridge Crystallographic Data Centre (CCDC), under deposition number CCDC 932131 and 932133. These data can be obtained free of charge from The Cambridge Crystallographic Data Centre via www.ccdc.cam.ac.uk/data_request/cif.

## Supplementary Material

Supplementary Figures, Supplementary Tables, Supplementary Methods and Supplementary References. Supplementary Figures 1-6, Supplementary Tables 1-7, Supplementary Methods and Supplementary References

Supplementary Data 1 Single crystal structure of 1•18H2O measured at 295 K

Supplementary Data 2 Single crystal structure of 1·ca.50H2O (single crystal structure of as prepared sample) measured at 100 K
