# Supplementary material for: A ferromagnetically coupled Fe42 cyanide-bridged nanocage
Source: Nat Commun. 2015 Jan 6;6:5955. doi: 10.1038/ncomms6955 (PMC4354210; doi:10.1038/ncomms6955)
Supplement: Supplementary Figures, Supplementary Tables, Supplementary Methods and Supplementary References. — Supplementary Figures 1-6, Supplementary Tables 1-7, Supplementary Methods and Supplementary References [file ncomms6955-s1.pdf]

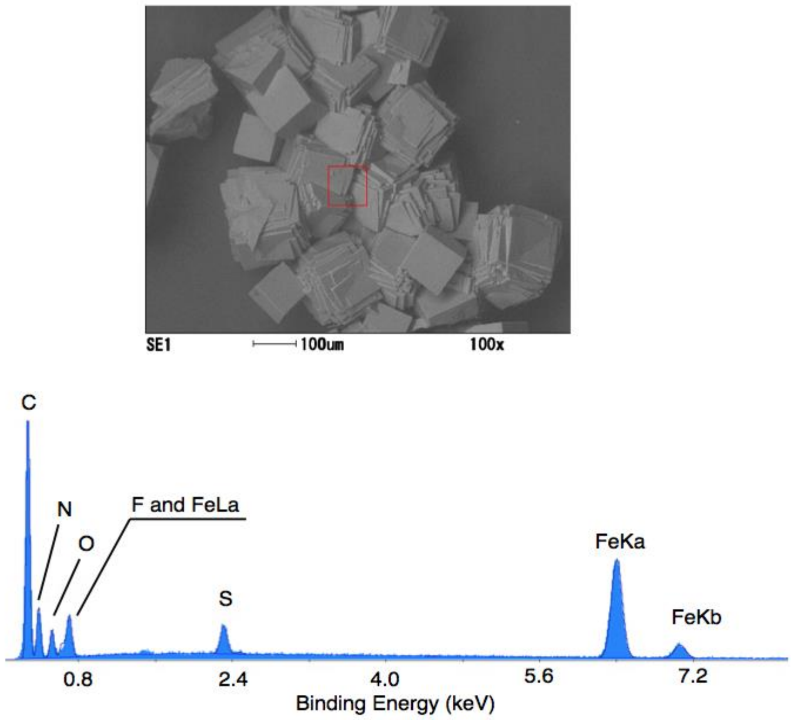


**Supplementary Figure 1** EDX spectrum of **1**·18$H_2O$, confirming the presence of $CF_3SO_3^-$ anion within **1**·18$H_2O$.

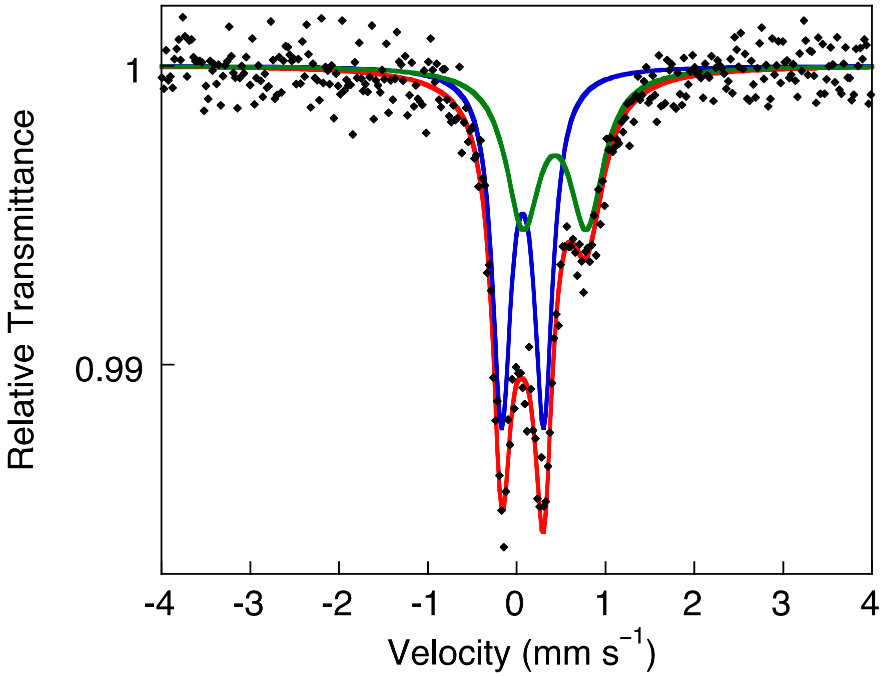


**Supplementary Figure 2** $^{57}$Fe Mössbauer spectrum of **1**·18$H_2O$ at 298 K (black square). Red line is fitted line composed of $Fe^{II}_{LS}$(blue line) and $Fe^{III}_{HS}$(green line).

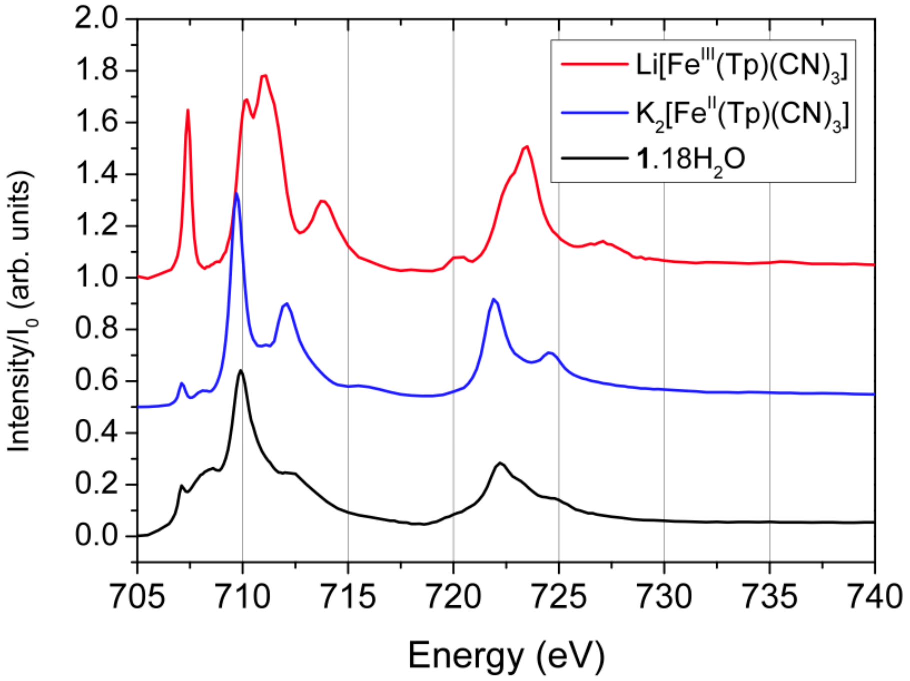


**Supplementary Figure 3** Fe L-edge XAS of **1**·18H$_2$O in comparison with Li[Fe$^{III}$(Tp)(CN)$_3$] and K$_2$[Fe$^{II}$(Tp)(CN)$_3$] measured at 200 K. From the line shape of the spectra it is clear that **1**·18H$_2$O exhibits a considerable contribution emanating from {Fe(Tp)(CN)$_3$}$^{2-}$ of low spin Fe(II) valence while the line shape of Li[Fe$^{III}$(Tp)(CN)$_3$] is not consistent with **1**·18H$_2$O. Supplementary Figure 3 shows the L-edge X-ray absorption spectroscopy (XAS) performed on **1**·18H$_2$O. The L-edge XAS probe from the 2p to the 3d Fe orbitals and is sensitive to the ligand field and metal-ligand covalence. Hence, the 24 {Fe(Tp)(CN)$_3$}, 12 {Fe(dpp)(H$_2$O)} and 6 {Fe(NC)$_4$(H$_2$O)$_2$} moieties all provide contributions which partially overlap resulting in the broadened spectral features observed. Typically ligand field multiplet calculations are employed to simulate the measured XAS spectra to identify metal site valence, crystal field splitting, orbital symmetries and the quantities of metal to ligand and ligand to metal covalence. In the case of **1**·18H$_2$O three separate simulations are necessary for the three different Fe moieties present before the ratio of their contributions can be determined. Such simulations are underway however the large number of parameters associated with the simulation of the broad **1**·18H$_2$O L-edge spectrum currently limits the merit of the simulation results. Alternatively some insight into the valence state composition of **1**·18H$_2$O may be deduced by comparison to reference Fe clusters. Comparison with the XAS of reference compounds Li[Fe$^{III}$(Tp)(CN)$_3$] and K$_2$[Fe$^{II}$(Tp)(CN)$_3$] some insight into the valence composition of the 24 {Fe(Tp)(CN)$_3$} can be inferred. The figure shows the three spectra corresponding

to the low spin Li[$Fe^{III}$(Tp)($CN)_3$] and $K_2$[$Fe^{II}$(Tp)($CN)_3$], plotted with respect to **1**·18$H_2O$. From the line shape of the spectra it is clear that **1**·18$H_2O$ exhibits a considerable contribution emanating from {Fe(Tp)($CN)_3$}$^{2-}$ of Fe(II) valence while the line shape of Li[$Fe^{III}$(Tp)($CN)_3$] is not consistent with **1**·18$H_2O$.

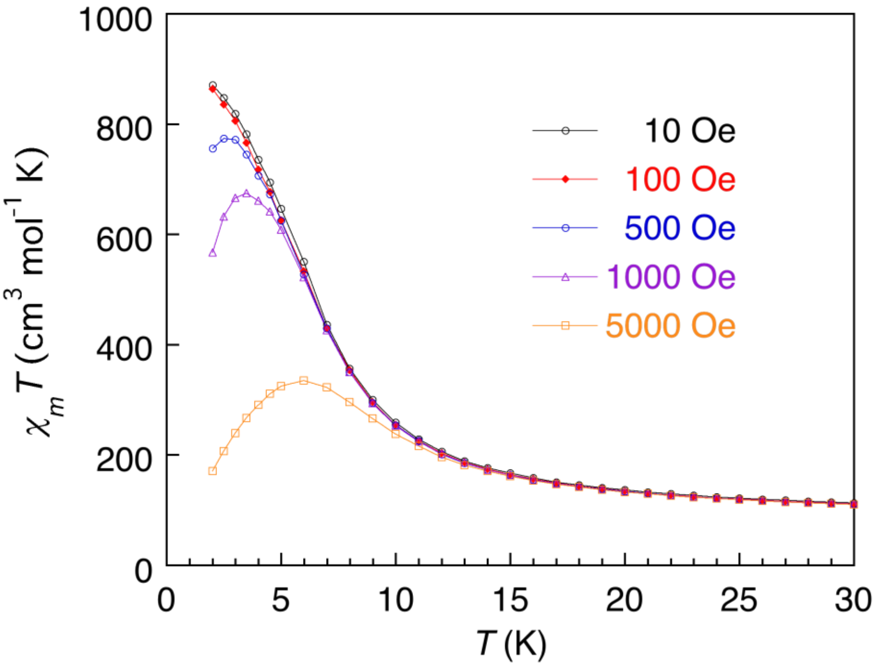


**Supplementary Figure 4** $\chi_m T(T)$ curve for **1**·18$H_2O$ between 2 to 30 K at 10, 100, 500, 1000 and 5000 Oe. The lines act as a guide the eye.

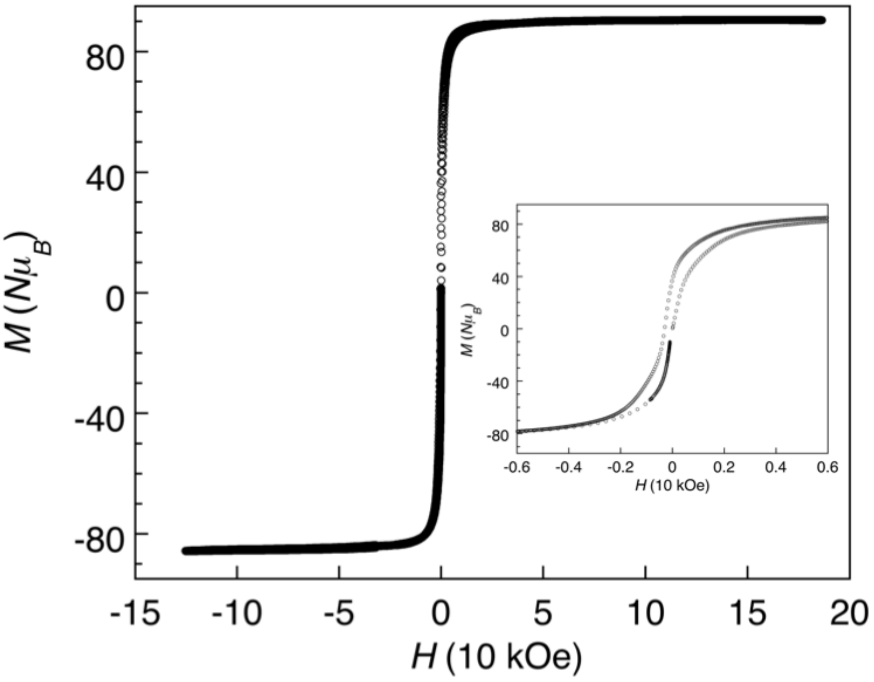


**Supplementary Figure 5** *M-H* plots for **1**·18$H_2O$ at high fields at 0.5 K. The magnetization *M* at 18 kOe is 90.4 $\mu_B$, which is in good agreement with the expected value of 90 $\mu_B$ (with $g$ = 2.0) for a ground state of $S_T$ = 90/2. The inset: expanded *M-H* plots for **1**·18$H_2O$. As described in the text, the temperature dependence of magnetization under various applied magnetic fields did not show evidence of spontaneous magnetization down to 0.5 K. Furthermore, no evidence of intermolecular interactions was observed in the EPR results when the temperature was decreased to 1.6 K. However, the inset indicates the possible presence of a discernible hysteresis loop at 0.5 K. Therefore, we intend to carefully investigate the magnetic properties at low temperatures using several methods, including heat capacity measurements.

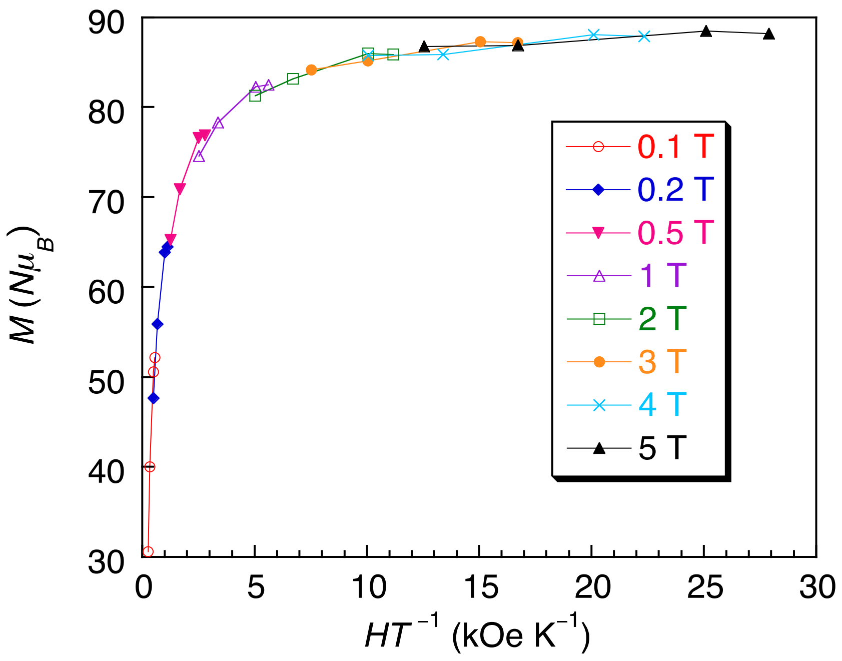


**Supplementary Figure 6** Plots of reduced magnetization ($M/N\mu_B$) vs $HT^{-1}$ in the range of 1.8 to 10 K for **1**·18$H_2O$.

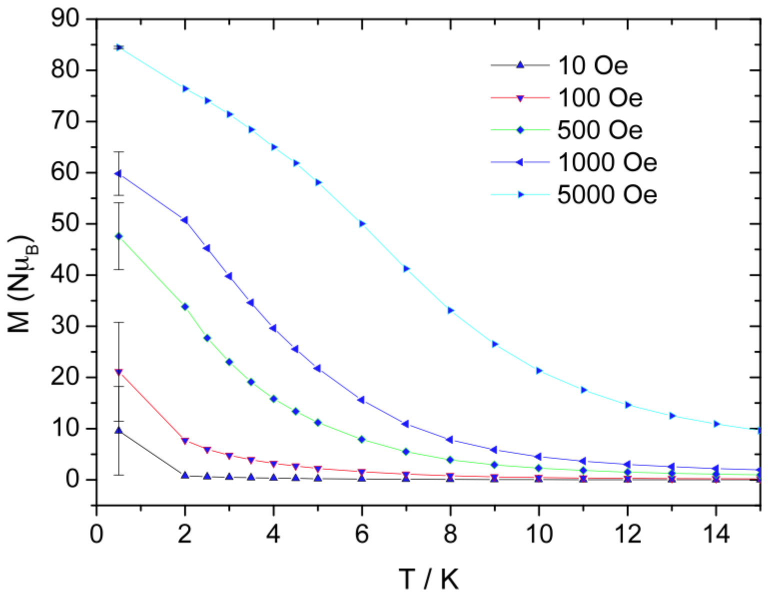


**Supplementary Figure 7** *M* versus *T* for various applied magnetic fields for **1**·18$H_2O$. Magnetization at 0.5 K originates from pulsed field magnetization data (c.f. Supplementary Fig. 5), the labelled uncertainties highlight the width of the measured magnetic hysteresis observed at 0.5 K.

**Supplementary Table 1:** Crystallographic Parameters for **1**·18$H_2O$, and **1**·ca.50$H_2O$

| | **1**·18$H_2O$ | **1**· ca. 50$H_2O$ |
|---|---|---|
| CCDC | CCDC 932131 | CCDC 932133 |
| $T$ / K | 295 | 100 |
| Formula | $C_{450}H_{492}B_{24}F_{18}Fe_{42}N_{240}O_{60}S_6$ | $C_{450}H_{548}B_{24}F_{18}Fe_{42}N_{240}O_{88}S_6$ |
| Space Group | *Pn-3n* | *Pn-3n* |
| $a$, Å | 30.8314(16) | 30.7679(13) |
| $b$, Å | 30.8314(16) | 30.7679(13) |
| $c$, Å | 30.8314(16) | 30.7679(13) |
| $\alpha$, ° | 90 | 90 |
| $\beta$, ° | 90 | 90 |
| $\gamma$, ° | 90 | 90 |
| $V$, Å$^3$ | 29308(3) | 29127(2) |
| $Z$ | 2 | 2 |
| $\mu$, mm$^{-1}$ | 0.714 | 0.765 |
| $\lambda$, Å | 0.6202 | 0.6186 |
| $2\theta$max | 47.5 | 47.5 |
| $R1$, $wR2$ [$I \geq 2\sigma(I)$] | 0.0722, 0.2085 | 0.0848, 0.2058 |
| $R1$, $wR2$ (all data) | 0.1243, 0.2391 | 0.0951, 0.2121 |
| GOF | 1.034 | 1.149 |

**Supplementary Table 2:** Selected bond distances (Å) and angles (°) for **1**·18$H_2O$ at 295K

| | | | |
|---|---|---|---|
| Fe(1)-N(4) | 2.008(4) | Fe(2)-N(3) | 2.014(5) |
| Fe(1)-N(6) | 2.014(4) | Fe(2)-O(1) | 1.979(11) |
| Fe(1)-N(8) | 2.001(4) | Fe(2)-O(2) | 2.341(17) |
| Fe(1)-C(1) | 1.866(5) | Fe(2)-N(3) | 2.014(5) |
| Fe(1)-C(2) | 1.894(5) | Fe(2)-N(3) | 2.014(5) |
| Fe(1)-C(3) | 1.862(6) | Fe(2)-N(3) | 2.014(5) |
| Fe(3)-N(1) | 2.030(5) | | |
| Fe(3)-N(2) | 2.083(4) | | |
| Fe(3)-N(10A) | 2.19(3) | | |
| Fe(3)-N(10B) | 2.164(16) | | |
| Fe(3)-O(3) | 2.110(7) | | |
| Fe(3)-N(1) | 2.030(5) | | |
| Fe(3)-N(2) | 2.083(4) | | |
| C(3)- Fe(1)-C(1) | 89.9(2) | O(1)-Fe(2)-N(3) | 97.59(18) |
| C(3)- Fe(1)-C(2) | 88.4(2) | O(1)-Fe(2)-N(3) | 97.59(18) |
| C(1)- Fe(1)-C(2) | 91.5(2) | N(3)-Fe(2)-N(3) | 164.8(4) |
| C(3)- Fe(1)-N(8) | 94.8(2) | O(1)-Fe(2)-N(3) | 97.59(18) |
| C(1)- Fe(1)-N(8) | 175.09(19) | N(3)-Fe(2)-N(3) | 89.00(5) |
| C(2)- Fe(1)-N(8) | 90.10(19) | N(3)-Fe(2)-N(3) | 89.00(5) |
| C(3)- Fe(1)-N(4) | 173.3(2) | O(1)-Fe(2)-N(3) | 97.59(18) |
| C(1)- Fe(1)-N(4) | 87.79(19) | N(3)-Fe(2)-N(3) | 89.00(5) |
| C(2)- Fe(1)-N(4) | 97.97(19) | N(3)-Fe(2)-N(3) | 89.00(5) |
| N(8)-Fe(1)-N(4) | 87.38(17) | N(3)-Fe(2)-N(3) | 164.8(4) |
| C(3)-Fe(1)-N(6) | 87.6(2) | O(1)-Fe(2)-O(2) | 180.000(1) |
| C(1)-Fe(1)-N(6) | 91.3(2) | N(3)-Fe(2)-O(2) | 82.41(18) |
| C(2)-Fe(1)-N(6) | 175.2(2) | N(3)-Fe(2)-O(2) | 82.41(18) |
| N(8)-Fe(1)-N(6) | 87.49(18) | N(3)-Fe(2)-O(2) | 82.41(18) |
| N(4)-Fe(1)-N(6) | 86.12(17) | N(3)-Fe(2)-O(2) | 82.41(18) |
| | | | |
| N(1)-Fe(3)-N(1) | 176.5(3) | N(1)-Fe(3)-N(10B) | 91.77(14) |
| N(1)-Fe(3)-N(2) | 89.43(17) | N(1)-Fe(3)-N(10B) | 91.77(14) |
| N(1)-Fe(3)-N(2) | 90.64(17) | N(2)-Fe(3)-N(10B) | 88.91(13) |
| N(1)-Fe(3)-N(2) | 90.64(17) | N(2)-Fe(3)-N(10B) | 88.91(13) |
| N(1)-Fe(3)-N(2) | 89.42(17) | O(3)-Fe(3)-N(10B) | 180.0(5) |
| N(2)-Fe(3)-N(2) | 177.8(3) | N(1)-Fe(3)-N(10A) | 91.77(14) |
| N(2)-Fe(3)-O(3) | 88.23(14) | N(1)-Fe(3)-N(10A) | 91.77(14) |
| N(2)-Fe(3)-O(3) | 88.23(14) | N(2)-Fe(3)-N(10A) | 88.91(13) |
| N(2)-Fe(3)-O(3) | 91.09(03) | N(2)-Fe(3)-N(10A) | 88.91(13) |
| N(2)-Fe(3)-O(3) | 91.09(03) | O(3)-Fe(3)-N(10A) | 180.0(7) |

**Supplementary Table 3:** Selected bond distances (Å) and angles (°) for **1**·ca 50$H_2O$ at 100 K

| | | | |
|---|---|---|---|
| Fe(1)-N(4) | 2.002(4) | Fe(2)-N(3) | 2.031(4) |
| Fe(1)-N(6) | 2.013(4) | Fe(2)-O(1) | 1.985(10) |
| Fe(1)-N(8) | 2.002(4) | Fe(2)-O(2) | 2.114(10) |
| Fe(1)-C(1) | 1.866(5) | Fe(2)-N(3) | 2.031(4) |
| Fe(1)-C(2) | 1.886(5) | Fe(2)-N(3) | 2.031(4) |
| Fe(1)-C(3) | 1.860(5) | Fe(2)-N(3) | 2.031(4) |
| Fe(3)-N(1) | 2.031(4) | | |
| Fe(3)-N(2) | 2.084(4) | | |
| Fe(3)-N(10A) | 2.15(2) | | |
| Fe(3)-N(10B) | 2.193(14) | | |
| Fe(3)-O(3) | 2.053(6) | | |
| Fe(3)-N(1) | 2.031(4) | | |
| Fe(3)-N(2) | 2.084(4) | | |
| C(3)-Fe(1)-C(1) | 89.9(2) | O(1)-Fe(2)-N(3) | 92.92(16) |
| C(3)-Fe(1)-C(2) | 88.1(2) | O(1)-Fe(2)-N(3) | 92.92(16) |
| C(1)-Fe(1)-C(2) | 92.3(2) | N(3)-Fe(2)-N(3) | 174.2(3) |
| C(3)-Fe(1)-N(8) | 94.31(19) | O(1)-Fe(2)-N(3) | 92.92(16) |
| C(1)-Fe(1)-N(8) | 175.49(18) | N(3)-Fe(2)-N(3) | 89.85(1) |
| C(2)-Fe(1)-N(8) | 89.52(19) | N(3)-Fe(2)-N(3) | 89.85(1) |
| C(3)-Fe(1)-N(4) | 173.9(2) | O(1)-Fe(2)-N(3) | 92.92(16) |
| C(1)-Fe(1)-N(4) | 88.24(18) | N(3)-Fe(2)-N(3) | 89.851(17) |
| C(2)-Fe(1)-N(4) | 97.83(19) | N(3)-Fe(2)-N(3) | 89.852(17) |
| N(8)-Fe(1)-N(4) | 87.44(16) | N(3)-Fe(2)-N(3) | 174.2(3) |
| C(3)-Fe(1)-N(6) | 87.6(2) | O(1)-Fe(2)-O(2) | 180.000(1) |
| C(1)-Fe(1)-N(6) | 90.70(19) | N(3)-Fe(2)-O(2) | 87.08(16) |
| C(2)-Fe(1)-N(6) | 174.74(19) | N(3)-Fe(2)-O(2) | 87.08(16) |
| N(8)-Fe(1)-N(6) | 87.77(18) | N(3)-Fe(2)-O(2) | 87.08(16) |
| N(4)-Fe(1)-N(6) | 86.56(17) | N(3)-Fe(2)-O(2) | 87.08(16) |
| | | | |
| N(1)-Fe(3)-N(1) | 177.7(3) | N(1)-Fe(3)-N(10A) | 91.16(12) |
| N(1)-Fe(3)-N(2) | 89.87(17) | N(1)-Fe(3)-N(10A) | 91.15(12) |
| N(1)-Fe(3)-N(2) | 90.18(17) | O(3)-Fe(3)-N(10A) | 180.0(6) |
| N(1)-Fe(3)-N(2) | 89.87(17) | N(2)-Fe(3)-N(10A) | 88.68(12) |
| N(1)-Fe(3)-N(2) | 90.18(17) | N(2)-Fe(3)-N(10A) | 88.68(12) |
| N(2)-Fe(3)-N(2) | 177.4(2) | N(1)-Fe(3)-N(10B) | 91.16(12) |
| N(1)-Fe(3)-O(3) | 88.84(12) | N(1)-Fe(3)-N(10B) | 91.15(12) |
| N(1)-Fe(3)-O(3) | 88.85(12) | O(3)- Fe(3)-N(10B) | 180.0(4) |
| N(1)-Fe(3)-N(2) | 89.87(17) | N(2)-Fe(3)-N(10B) | 88.68(12) |
| O(3)-Fe(3)-N(2) | 91.32(12) | N(2)-Fe(3)-N(10B) | 88.68(12) |

**Supplementary Table 4:** Mössbauer parameters for **1**·18$H_2O$ at 298 K (See Supplementary Fig. 1)

| | $Fe^{II}_{LS}$ | $Fe^{III}_{HS}$ |
|---|---|---|
| $\delta$ (mm $s^{-1}$) | 0.0654 | 0.428 |
| $\Delta E_Q$ (mm $s^{-1}$) | 0.471 | 0.713 |
| Content | 0.55 | 0.45 |
| Calcd. | 0.57 | 0.43 |

**Supplementary Table 5:** Cartesian coordinates of $[\{Fe(Tp)(CN)_3\}_2\{Fe(CN)_2(H_2O)_2\}\{Fe(CN)_2(4\text{-methylpyridine})(H_2O)\}]^{2-}$ in the HSFM state.

| Atom | Coordinates (Angstroms) X | Y | Z |
|---|---|---|---|
| N | -3.960988 | -0.208146 | 1.817812 |
| C | -3.146965 | -0.366585 | 2.869253 |
| C | -3.892648 | -0.368476 | 4.070110 |
| C | -5.216154 | -0.202690 | 3.656460 |
| N | -5.230355 | -0.109098 | 2.304062 |
| B | -6.399609 | 0.066066 | 1.304569 |
| N | -6.373112 | -1.135931 | 0.338664 |
| C | -7.300500 | -2.088609 | 0.070237 |
| C | -6.762072 | -2.971285 | -0.867308 |
| C | -5.465918 | -2.468101 | -1.120273 |
| N | -5.245614 | -1.366269 | -0.392729 |
| Fe | -3.649941 | -0.122856 | -0.190958 |
| N | -4.966325 | 1.436550 | -0.220429 |
| C | -4.951102 | 2.644761 | -0.799474 |
| C | -6.126062 | 3.357614 | -0.468900 |
| C | -6.844205 | 2.488259 | 0.353497 |
| N | -6.128639 | 1.343508 | 0.488102 |
| C | -2.462171 | -1.558350 | -0.138059 |
| N | -1.690918 | -2.454242 | -0.117147 |
| Fe | -0.267154 | -3.879987 | -0.131519 |
| N | -1.704910 | -5.316473 | -0.459172 |
| C | -2.525932 | -6.165839 | -0.560743 |
| N | 1.068081 | -2.555752 | 0.648260 |
| C | 1.965790 | -1.816523 | 0.421765 |
| Fe | 3.372631 | -0.786389 | -0.255696 |
| N | 4.735930 | -2.205438 | 0.284506 |
| C | 4.728150 | -3.544729 | 0.267211 |
| C | 5.949365 | -4.044902 | 0.774722 |
| C | 6.688153 | -2.906338 | 1.098436 |
| N | 5.939777 | -1.815367 | 0.795958 |
| B | 6.221267 | -0.312158 | 0.965978 |
| N | 5.116288 | 0.271262 | 1.882232 |
| C | 5.190107 | 0.909390 | 3.076175 |
| C | 3.891344 | 1.214990 | 3.489734 |
| C | 3.067150 | 0.710089 | 2.457729 |

| | | | |
|---|---|---|---|
| N | 3.814227 | 0.146018 | 1.500242 |
| N | 6.088322 | 0.363066 | -0.414967 |
| C | 6.962446 | 1.110635 | -1.134331 |
| C | 6.333908 | 1.498224 | -2.319047 |
| C | 5.043230 | 0.927208 | -2.236842 |
| N | 4.909627 | 0.247951 | -1.090614 |
| C | 2.160232 | 0.551831 | -0.749925 |
| N | 1.398399 | 1.395943 | -1.067995 |
| Fe | -0.003816 | 2.779874 | -1.561194 |
| N | -1.320596 | 1.832142 | -0.295078 |
| C | -2.210397 | 1.071685 | -0.121522 |
| N | 1.276442 | 3.692544 | -2.860747 |
| C | 2.018506 | 4.229314 | -3.613057 |
| N | -1.422744 | 4.238291 | -1.854462 |
| C | -2.238815 | 5.081715 | -2.014704 |
| N | 0.773085 | 4.031194 | 0.110338 |
| C | 2.091665 | 4.264092 | 0.240162 |
| C | 2.619862 | 5.010487 | 1.296031 |
| C | 1.757732 | 5.545807 | 2.270544 |
| C | 0.384391 | 5.295236 | 2.117011 |
| C | -0.065047 | 4.538236 | 1.028295 |
| C | 2.295244 | 6.350690 | 3.432144 |
| O | -0.730069 | 1.674293 | -3.215431 |
| C | 2.965148 | -1.736497 | -1.860721 |
| N | 2.572139 | -2.408764 | -2.751578 |
| O | 0.152287 | -3.407611 | -2.118112 |
| O | -0.656002 | -4.639182 | 2.008939 |
| N | 1.179864 | -5.337322 | -0.044305 |
| C | 1.980135 | -6.194239 | 0.129593 |
| C | -3.365963 | 0.027564 | -2.075134 |
| N | -3.050123 | 0.228249 | -3.197865 |
| H | -1.595333 | 1.153809 | -3.184153 |
| H | -0.771767 | 2.257719 | -3.989858 |
| H | -0.406861 | -2.652947 | -2.374380 |
| H | 1.101952 | -3.131576 | -2.365869 |
| H | -1.356945 | -5.245163 | 1.696572 |
| H | 0.155787 | -5.181755 | 2.007235 |
| H | 1.487299 | 6.691364 | 4.090882 |
| H | 2.995545 | 5.750465 | 4.029749 |
| H | -1.122076 | 4.327531 | 0.874288 |
| H | -0.341204 | 5.680307 | 2.833251 |
| H | 3.698105 | 5.159027 | 1.354358 |

| | | | |
|---|---|---|---|
| H | 2.728847 | 3.833960 | -0.530695 |
| H | -8.266262 | -2.071732 | 0.563764 |
| H | -7.223512 | -3.853463 | -1.294167 |
| H | -4.689507 | -2.849803 | -1.773356 |
| H | -7.802457 | 2.599192 | 0.849789 |
| H | -6.395863 | 4.358276 | -0.783903 |
| H | -4.109823 | 2.955986 | -1.409170 |
| H | -6.139945 | -0.148039 | 4.222228 |
| H | -3.521321 | -0.484138 | 5.081337 |
| H | -2.080176 | -0.483069 | 2.712726 |
| H | 2.843400 | 7.234102 | 3.075816 |
| H | 7.958708 | 1.310586 | -0.754698 |
| H | 6.736956 | 2.106417 | -3.119792 |
| H | 4.214859 | 0.981055 | -2.934469 |
| H | 1.987459 | 0.726760 | 2.357881 |
| H | 3.583773 | 1.719150 | 4.398193 |
| H | 6.151442 | 1.095890 | 3.542654 |
| H | 3.860932 | -4.088022 | -0.092764 |
| H | 6.236459 | -5.083361 | 0.886875 |
| H | 7.682181 | -2.792283 | 1.517769 |
| H | 7.315049 | -0.121631 | 1.438722 |
| H | -7.455095 | 0.133775 | 1.884824 |

**Supplementary Table 6:** Cartesian coordinates of [{Fe(Tp)(CN)$_3$}$_2${Fe(CN)$_2$(H$_2$O)$_2$}{Fe(CN)$_2$(4-methylpyridine)(H$_2$O)}]$^{2-}$ in the LSFM state.

| Atom | Coordinates (Angstroms) | | |
|---|---|---|---|
| | X | Y | Z |
| N | -3.882433 | -0.143221 | 1.796987 |
| C | -3.071858 | -0.258502 | 2.856652 |
| C | -3.822041 | -0.225378 | 4.055141 |
| C | -5.145347 | -0.084458 | 3.631230 |
| N | -5.153889 | -0.038023 | 2.275930 |
| B | -6.319377 | 0.096678 | 1.265685 |
| N | -6.280849 | -1.134662 | 0.338241 |
| C | -7.203043 | -2.100398 | 0.096948 |
| C | -6.656841 | -3.008269 | -0.811410 |
| C | -5.362261 | -2.505385 | -1.076115 |
| N | -5.149546 | -1.380194 | -0.383009 |
| Fe | -3.565928 | -0.120258 | -0.212512 |
| N | -4.895201 | 1.424832 | -0.303846 |
| C | -4.894693 | 2.608438 | -0.931463 |
| C | -6.076554 | 3.322933 | -0.627371 |
| C | -6.784204 | 2.480394 | 0.231090 |
| N | -6.055338 | 1.349727 | 0.410335 |
| C | -2.359840 | -1.565990 | -0.104834 |
| N | -1.602650 | -2.466479 | -0.069501 |
| Fe | -0.304055 | -3.877445 | -0.074160 |
| N | -1.706413 | -5.132354 | -0.497801 |
| C | -2.574789 | -5.920153 | -0.657435 |
| N | 1.056543 | -2.627416 | 0.457035 |
| C | 1.934199 | -1.857481 | 0.281456 |
| Fe | 3.348108 | -0.761202 | -0.300453 |
| N | 4.728022 | -2.161367 | 0.238871 |
| C | 4.752555 | -3.499470 | 0.187461 |
| C | 5.970981 | -3.985507 | 0.716143 |
| C | 6.673919 | -2.839265 | 1.088973 |
| N | 5.907850 | -1.758358 | 0.793580 |
| B | 6.146673 | -0.253757 | 1.012541 |
| N | 5.003072 | 0.274609 | 1.915499 |
| C | 5.029485 | 0.873351 | 3.131896 |
| C | 3.712662 | 1.130921 | 3.520545 |
| C | 2.929406 | 0.641406 | 2.449307 |
| N | 3.715209 | 0.129056 | 1.493779 |

| | | | |
|---|---|---|---|
| N | 6.033810 | 0.458544 | -0.350434 |
| C | 6.905781 | 1.255233 | -1.018939 |
| C | 6.297218 | 1.669272 | -2.204862 |
| C | 5.020349 | 1.062135 | -2.177202 |
| N | 4.875523 | 0.337900 | -1.060336 |
| C | 2.106291 | 0.578677 | -0.793236 |
| N | 1.341813 | 1.419450 | -1.094562 |
| Fe | 0.060058 | 2.786070 | -1.522998 |
| N | -1.233710 | 1.830902 | -0.440075 |
| C | -2.119392 | 1.091458 | -0.199767 |
| N | 1.323315 | 3.706962 | -2.641672 |
| C | 2.101898 | 4.263529 | -3.335504 |
| N | -1.228803 | 4.164111 | -1.903297 |
| C | -2.033827 | 5.000808 | -2.124819 |
| N | 0.675618 | 3.822189 | 0.077963 |
| C | 1.988867 | 4.037240 | 0.292859 |
| C | 2.451687 | 4.736831 | 1.407809 |
| C | 1.539108 | 5.246657 | 2.347443 |
| C | 0.176794 | 5.012003 | 2.102061 |
| C | -0.216270 | 4.300122 | 0.965134 |
| C | 2.009046 | 6.019657 | 3.558246 |
| O | -0.537729 | 1.795910 | -3.120558 |
| C | 2.994609 | -1.683657 | -1.935185 |
| N | 2.653326 | -2.357177 | -2.847474 |
| O | 0.047106 | -3.528861 | -1.962427 |
| O | -0.713430 | -4.364731 | 1.856062 |
| N | 0.998215 | -5.294941 | 0.030460 |
| C | 1.764258 | -6.177910 | 0.211966 |
| C | -3.227982 | -0.021870 | -2.090386 |
| N | -2.841654 | 0.164530 | -3.195280 |
| H | -1.374381 | 1.213670 | -3.085076 |
| H | -0.691719 | 2.456536 | -3.816706 |
| H | -0.575869 | -2.836829 | -2.248579 |
| H | 0.976044 | -3.195317 | -2.237832 |
| H | -1.531887 | -4.877110 | 1.698683 |
| H | -0.006953 | -5.032189 | 1.972567 |
| H | 1.195892 | 6.165726 | 4.279544 |
| H | 2.831491 | 5.495659 | 4.063269 |
| H | -1.261159 | 4.099764 | 0.746059 |
| H | -0.590186 | 5.373811 | 2.786227 |
| H | 3.526003 | 4.866211 | 1.535367 |
| H | 2.667359 | 3.631361 | -0.451731 |

| | | | |
|---|---|---|---|
| H | -8.170541 | -2.072813 | 0.586685 |
| H | -7.111965 | -3.905642 | -1.212816 |
| H | -4.581297 | -2.904512 | -1.713207 |
| H | -7.743566 | 2.600222 | 0.723202 |
| H | -6.358900 | 4.306540 | -0.982915 |
| H | -4.058510 | 2.901301 | -1.556931 |
| H | -6.071918 | -0.016783 | 4.191094 |
| H | -3.453929 | -0.300201 | 5.071554 |
| H | -2.003576 | -0.369620 | 2.706314 |
| H | 2.382869 | 7.011038 | 3.263583 |
| H | 7.886084 | 1.467362 | -0.605759 |
| H | 6.702743 | 2.318409 | -2.971519 |
| H | 4.207802 | 1.122804 | -2.892619 |
| H | 1.852714 | 0.633624 | 2.320172 |
| H | 3.368412 | 1.594562 | 4.437536 |
| H | 5.973175 | 1.067220 | 3.630370 |
| H | 3.907381 | -4.050143 | -0.211444 |
| H | 6.280386 | -5.019456 | 0.810150 |
| H | 7.653148 | -2.713452 | 1.538689 |
| H | 7.222390 | -0.050325 | 1.520980 |
| H | -7.378195 | 0.174563 | 1.839828 |

**Supplementary Table 7:** Cartesian coordinates of $[\{Fe(Tp)(CN)_3\}_2\{Fe(CN)_2(H_2O)_2\}\{Fe(CN)_2(4\text{-methylpyridine})(H_2O)\}]^{2-}$ in the LSAF state.

| Atom | Coordinates (Angstroms) | | |
|---|---|---|---|
| | X | Y | Z |
| N | 3.903673 | 0.150795 | 1.804120 |
| C | 3.103806 | 0.247178 | 2.873672 |
| C | 3.867980 | 0.218282 | 4.063392 |
| C | 5.188293 | 0.100218 | 3.623449 |
| N | 5.182031 | 0.061874 | 2.267987 |
| B | 6.338072 | -0.049669 | 1.243775 |
| N | 6.270929 | 1.185593 | 0.324024 |
| C | 7.172560 | 2.170146 | 0.081391 |
| C | 6.600692 | 3.074695 | -0.814372 |
| C | 5.313248 | 2.549988 | -1.070968 |
| N | 5.128339 | 1.415758 | -0.384532 |
| Fe | 3.564100 | 0.131337 | -0.202298 |
| N | 4.914431 | -1.391886 | -0.314883 |
| C | 4.923714 | -2.572670 | -0.947767 |
| C | 6.120232 | -3.270160 | -0.662437 |
| C | 6.826241 | -2.420294 | 0.190243 |
| N | 6.082585 | -1.301943 | 0.384228 |
| C | 2.348539 | 1.567838 | -0.086616 |
| N | 1.583886 | 2.461895 | -0.050118 |
| Fe | 0.267309 | 3.857014 | -0.042395 |
| N | 1.639589 | 5.127646 | -0.509519 |
| C | 2.488866 | 5.930989 | -0.693717 |
| N | -1.052664 | 2.586050 | 0.525180 |
| C | -1.936919 | 1.817320 | 0.385403 |
| Fe | -3.339681 | 0.748510 | -0.261656 |
| N | -4.719774 | 2.154502 | 0.262983 |
| C | -4.727306 | 3.493556 | 0.238019 |
| C | -5.957019 | 3.983764 | 0.735513 |
| C | -6.685310 | 2.838860 | 1.060963 |
| N | -5.922480 | 1.754909 | 0.769081 |
| B | -6.189469 | 0.249038 | 0.940868 |
| N | -5.085724 | -0.319462 | 1.868327 |
| C | -5.163107 | -0.951659 | 3.065419 |
| C | -3.864622 | -1.242065 | 3.490796 |
| C | -3.037627 | -0.734998 | 2.461509 |
| N | -3.782068 | -0.183117 | 1.494872 |

| | | | |
|---|---|---|---|
| N | -6.039394 | -0.428641 | -0.436418 |
| C | -6.899823 | -1.189804 | -1.158793 |
| C | -6.257083 | -1.577446 | -2.335635 |
| C | -4.972824 | -0.992630 | -2.245749 |
| N | -4.855839 | -0.304946 | -1.102774 |
| C | -2.099455 | -0.591894 | -0.742906 |
| N | -1.334894 | -1.433965 | -1.042735 |
| Fe | -0.042241 | -2.776519 | -1.489043 |
| N | 1.262056 | -1.839713 | -0.425340 |
| C | 2.138339 | -1.091707 | -0.173242 |
| N | -1.302550 | -3.680594 | -2.612660 |
| C | -2.073585 | -4.234649 | -3.317711 |
| N | 1.287874 | -4.090332 | -1.937214 |
| C | 2.107569 | -4.896241 | -2.213854 |
| N | -0.649585 | -3.850336 | 0.071898 |
| C | -1.960910 | -4.041248 | 0.318772 |
| C | -2.413780 | -4.777036 | 1.415082 |
| C | -1.491378 | -5.353318 | 2.304984 |
| C | -0.130183 | -5.142343 | 2.031769 |
| C | 0.249680 | -4.392194 | 0.915216 |
| C | -1.947190 | -6.175711 | 3.488520 |
| O | 0.537966 | -1.711282 | -3.099347 |
| C | -2.913081 | 1.704615 | -1.859870 |
| N | -2.491647 | 2.399942 | -2.720661 |
| O | -0.112082 | 3.435867 | -1.922764 |
| O | 0.694105 | 4.417948 | 1.869530 |
| N | -1.055834 | 5.252955 | 0.058523 |
| C | -1.833886 | 6.126403 | 0.235843 |
| C | 3.216244 | 0.040048 | -2.081079 |
| N | 2.833158 | -0.137233 | -3.187794 |
| H | 1.356708 | -1.130900 | -3.018605 |
| H | 0.756126 | -2.365248 | -3.784016 |
| H | 0.476631 | 2.699130 | -2.169617 |
| H | -1.051017 | 3.121573 | -2.164019 |
| H | 1.486123 | 4.956836 | 1.667893 |
| H | -0.036757 | 5.062068 | 1.969083 |
| H | -1.152887 | -6.269829 | 4.239230 |
| H | -2.827034 | -5.726041 | 3.966848 |
| H | 1.294019 | -4.211204 | 0.677266 |
| H | 0.645184 | -5.552104 | 2.678607 |
| H | -3.487473 | -4.882620 | 1.567867 |
| H | -2.649490 | -3.584326 | -0.385620 |

| | | | |
|---|---|---|---|
| H | 8.145412 | 2.156599 | 0.561044 |
| H | 7.034872 | 3.983397 | -1.213410 |
| H | 4.519446 | 2.939510 | -1.698022 |
| H | 7.794038 | -2.527258 | 0.668597 |
| H | 6.413123 | -4.247696 | -1.026044 |
| H | 4.084409 | -2.874344 | -1.564882 |
| H | 6.122130 | 0.043326 | 4.172343 |
| H | 3.510564 | 0.281463 | 5.084394 |
| H | 2.032193 | 0.341573 | 2.736228 |
| H | -2.228455 | -7.189753 | 3.167966 |
| H | -7.896807 | -1.398839 | -0.785923 |
| H | -6.645722 | -2.196385 | -3.135272 |
| H | -4.137501 | -1.043264 | -2.935296 |
| H | -1.957168 | -0.742164 | 2.369456 |
| H | -3.559363 | -1.738176 | 4.404557 |
| H | -6.126269 | -1.144970 | 3.525372 |
| H | -3.863694 | 4.042249 | -0.121934 |
| H | -6.257300 | 5.019370 | 0.840041 |
| H | -7.680931 | 2.715974 | 1.474010 |
| H | -7.285018 | 0.048905 | 1.406333 |
| H | 7.404171 | -0.116094 | 1.805669 |

**Supplementary Methods**.

**DFT calculation: Computational method.**

The structure of $[\{Fe(Tp)(CN)_3\}_2\{Fe(CN)_2(H_2O)_2\}\{Fe(CN)_2(4\text{-methylpyridine})(H_2O)\}]^{2-}$ was optimized by using the B3LYP* functional.[1-4] The Wachters-Hay basis set[5,6] was used for Fe atoms and the D95** basis set[7,8] for H, B, C, N, and O atoms. The program we used is Gaussian 09.[9] Spin multiplicities of the high spin ferromagnetic (HSFM) state, the low spin ferromagnetic (LSFM) state, and the low spin antiferromagnetic (LSAF) state are the undecet state, the triplet state, and the open-shell singlet state, respectively. The high spin antiferromagnetic (HSAF) state is not available as a low-lying open-shell singlet state. Time-dependent density functional theory (TDDFT)[10,11] has been used to calculate the excited-state energies. An electron density difference map (EDDM)[12] is a representation of the changes in electron density that occur for a given electronic transition. It is calculated using the information on the single-excited configurations that contribute to each transition. The relative contribution is based on the square of the configuration's coefficient.

**Exchange coupling.** The strength of exchange coupling interaction in the $[Fe^{III}(HS)\text{- }Fe^{III}(HS)]$ state evaluated for optimized structures using BS methodology. The scheme proposed by Yamaguchi and co-workers[13,14] is used:

$$J = \frac{2\left(E_{BS} - E_{HS}\right)}{\left\langle S^2 \right\rangle_{HS} - \left\langle S^2 \right\rangle_{BS}} \qquad (1)$$

where $E_X$ and $\langle S^2 \rangle_X$ denote the total energy and total angular momentum of the spin state X (X = BS (LSAF) and HS (HSAF)). *J* is approximately spin-projected (AP) procedure.

**Supplementary References:**


(1) Becke, A. D. Density-functional exchange-energy approximation with correct asymptotic behavior, *Phys. Rev. A* **38**, *38*, 3098-3100 (1998).

(2) Lee, C., Yang, W. & Parr, R. G. Development of the Colle-Salvetti correlation-energy formula into a functional of the electron density, *Phys. Rev. B* **37**, 785-789 (1988).

(3) Becke, A. D. Density-functional thermochemistry. III. The role of exact exchange *J. Chem. Phys.* **98**, 5648-5652 (1993).

(4) Reiher, M. Theoretical Study of the $Fe(phen)_2(NCS)_2$ Spin-Crossover Complex with Reparametrized Density Functionals, *Inorg. Chem*. **41**, 6928-6935 (2002).

(5) Wachters, A. J. H. Gaussian Basis Set for Molecular Wavefunctions Containing Third-Row Atoms, *J. Chem. Phys.* **52**, 1033-1036 (1970).

(6) Hay, P. J. Gaussian basis sets for molecular calculations. The representation of 3d orbitals in transition‐metal atoms, *J. Chem. Phys.* **66**, 4377-4384 (1977).

(7) Raghavachari, K. & Trucks, G. W. Highly correlated systems. Excitation energies of first row transition metals Sc–Cu, *J. Chem. Phys.* **91**, 1062 (1989).

(8) Dunning, T. H. & Hay, P. J. In *Modern Theoretical Chemistry*; Schaefer, H. F., III, Ed.; Plenum: New York, Vol. 3, pp 1-27 (1976).

(9) Gaussian 09 (Revision A.02), M. J. Frisch, G. W. Trucks, H. B. Schlegel, G. E. Scuseria, M. A. Robb, J. R. Cheeseman, G. Scalmani, V. Barone, B. Mennucci, G. A. Petersson, H. Nakatsuji, M. Caricato, X. Li, H. P. Hratchian, A. F. Izmaylov, J. Bloino, G. Zheng, J. L. Sonnenberg, M. Hada, M. Ehara, K. Toyota, R. Fukuda, J. Hasegawa, M. Ishida, T. Nakajima, Y. Honda, O. Kitao, H. Nakai, T. Vreven, J. A. Montgomery, Jr., J. E. Peralta, F. Ogliaro, M. Bearpark, J. J. Heyd, E. Brothers, K. N. Kudin, V. N. Staroverov, R. Kobayashi, J. Normand, K. Raghavachari, A. Rendell, J. C. Burant, S. S. Iyengar, J. Tomasi, M. Cossi, N. Rega, J. M. Millam, M. Klene, J. E. Knox, J. B. Cross, V. Bakken, C. Adamo, J. Jaramillo, R. Gomperts, R. E. Stratmann, O. Yazyev, A. J. Austin, R. Cammi, C. Pomelli, J. W. Ochterski, R. L. Martin, K. Morokuma, V. G. Zakrzewski, G. A. Voth, P. Salvador, J. J. Dannenberg, S. Dapprich, A. D. Daniels, Ö. Farkas, J. B. Foresman, J. V. Ortiz, J. Cioslowski, and D. J. Fox, Gaussian, Inc., Wallingford CT, **2009**.

(10) Gross, E. K. U., Dobson, J. F. & Petersilka, M. *In Density Functional Theory*; Nalewajski, R. F., Ed.; Springer: Heidelberg (1996).

(11) Casida, M. E. *In Recent Advances in Density Functional Methods*; Chong, D. P., Ed.; World Scientific: Singapore, Vol. 1, pp 155-193 (1995).

(12) Browne, W. R., O'Boyle, N. M., McGarvey, J. J. & Vos, J. G. Elucidating excited state electronic

structure and intercomponent interactions in multicomponent and supramolecular systems, *Chem. Soc. Rev.* **34**, 641-663 (2005).

(13) Soda, T. *et al.* Ab initio computations of effective exchange integrals for H–H, H–He–H and $Mn_2O_2$ complex: comparison of broken-symmetry approaches, *Chem. Phys. Lett.* **319**, 223-230 (2000).

(14) Shoji, M. *et al.* A general algorithm for calculation of Heisenberg exchange integral J in multispin systems, *Chem. Phys. Lett.* **432**, 343-347 (2006).
